# Phikud Navakot Modulates the Level of Pro-Inflammatory Mediators and the Protein Expression of SOD1 and 2 and the Nrf2/HO-1 Signaling Pathway in Rats with Acute Myocardial Infarction

**DOI:** 10.1155/2019/4823645

**Published:** 2019-09-18

**Authors:** Orapin Gerdprasert, Nantana Choomchuay, Boonrat Chantong, Narueporn Sutanthavibul, Duangdeun Meksuriyen, Punnee Nusuetrong

**Affiliations:** ^1^Faculty of Medicine, Srinakharinwirot University, Bangkok 10110, Thailand; ^2^Faculty of Veterinary Science, Mahidol University, Nakorn Pathom 73170, Thailand; ^3^Faculty of Pharmaceutical Sciences, Chulalongkorn University, Bangkok 10330, Thailand; ^4^College of Pharmacy, Rangsit University, Bangkok, Pathum Thani 10200, Thailand

## Abstract

Phikud Navakot (PN) is nine major herbs in a famous traditional Thai recipe namely “Yahom Navakot” used to treat cardiovascular disorders. This study investigated the cardioprotective effects of PN formula on isoproterenol-induced myocardial infarction (IMI) in Sprague-Dawley rats. Forty-five rats were randomly divided into nine groups (*n* = 5 per group): the control, the IMI, the IMI + propranolol, the control or the IMI + PN formula (PN ethanolic extract at doses of 64, 127, or 255 mg/kg) by oroesophageal gavage for 28 days. The ST segment and serum troponin T levels were significantly increased in IMI rats. PN did not eliminate tissue necrosis, infiltration of inflammatory cells, or interstitial edema in IMI rats. All doses of PN decreased (*p* < 0.001) serum TNF-*α* and IL-6 levels. PN (127 and 255 mg/kg) up-regulated (*p* < 0.05) heme oxygenase (HO)-1 expression, whereas PN (255 mg/kg) significantly increased superoxide dismutase (SOD) 1 and 2 expression, compared with IMI rats. Nuclear factor erythroid 2-related factor 2 (Nrf2) and HO-1 expression significantly increased in IMI rats and IMI rats that received PN. PN formula possesses potential anti-inflammatory and antioxidant properties by modulating the levels of TNF-*α*, IL-6 and antioxidant enzymes. Our study reveals a novel cardioprotective effect of PN in IMI rats through the Nrf2/HO-1 signaling.

## 1. Introduction

Myocardial infarction (MI) is a major cause of death worldwide and puts surviving patients at risk of developing other vascular diseases. Research into the potential of new therapeutic agents to treat MI has been facilitated by the use of animal models. For instance, the administration of isoproterenol (ISO), a *β*‐adrenergic receptor agonist, was used to mimic the characteristics of MI in rats to study the effectiveness of medicinal plants or active compounds in disease intervention [1, 2]. ISO administration also causes an elevation in the ST segment in ECG readings. Furthermore, it caused an increase in the levels of cardiac injury biomarkers, including troponin I, troponin T, creatine kinase-MB, lactate dehydrogenase, alkaline phosphatase, serum glutamic oxaloacetic transaminase, aspartate transaminase, and alanine transaminase [1–4]. ISO-treated rats exhibited myocardial cell swelling, myocardial degeneration, loss of myofibrils, and diffuse infiltration of inflammatory cells [1, 2, 4]. There was also an increase in cardiac tissues and serum levels of the pro-inflammatory cytokines interleukin-6 (IL-6) and tumor necrosis factor-*α* (TNF-*α*) [1, 4]. Moreover, the ISO-induced myocardial changes were the result of an increase in oxidative stress through reductions of the components of the myocardial antioxidant system, including glutathione, glutathione reductase, glutathione-S-transferase, glutathione peroxidase (GPx), superoxide dismutase (SOD), and catalase, in rat hearts [3, 5]. Furthermore, it was shown that a reduction in heme oxygenase (HO)-1 levels and an induction of endogenous antioxidants occurred *via* the activity of nuclear factor erythroid 2-related factor 2 (Nrf2) [5]. In contrast, ISO was reported to cause an increase in the expression of Nrf2 and HO-1 [6]. Other compounds associated with MI are nitric oxide (NO) and its metabolites, which have been reported to protect the heart from ischemia/reperfusion (IR) injury and decrease MI in general [7].

Phikud Navakot (PN), a major component of “Yahom Navakot,” is composed of an equal amount of nine herbs, namely *Angelica dahurica* (Fisch.) Benth. & Hook. f. (Apiaceae) root, *Angelica sinensis* (Oliv.) Diels (Apiaceae) root, *Atractylodes lancea* (Thunb.) DC. (Asteraceae) rhizome, *Ligusticum chuanxiong* Hort. (Apiaceae) rhizome, *Artemisia pallens* Walls ex DC. (Asteraceae) aerial part, *Saussurea costus* (Falc.) Lipsch. (Asteraceae) rhizome, *Picrorhiza kurrooa* Royle ex Benth. (Scrophulariaceae) rhizome, *Terminalia chebula* Retz. (Combretaceae) gall, and *Nardostachys jatamansi* (D. Don) DC. (Valerianaceae) root and rhizome. “Yahom Navakot” itself is a Thai herbal formula that has been traditionally used for the treatment of circulatory disorders and is included in the National List of Essential Medicine of Thailand 2013. Recently, PN and some of its components, such as *T*. *chebula*, *P*. *kurrooa*, *A*. *pallen*, and *N*. *jatamansi*, were shown to possess free radical scavenging activities against superoxide anions and hydroxyl radicals in an *in vitro* study [8]. Due to this finding and the fact that oxidative stress and inflammation are the major causes of cardiac injury in MI [9, 10], this study evaluated the cardioprotective effects of PN in ISO-induced MI (IMI) in rats using various techniques. These techniques included an assessment of the degree of histopathological changes, measurements of levels of NO, and the proinflammatory cytokines TNF-*α* and IL-6, and the evaluation of the protein expression of the antioxidant enzymes GPx, SOD, catalase, Nrf2, and HO-1.

## 2. Materials and Methods

### 2.1. Reagents and Chemicals

Isoproterenol hydrochloride, propranolol (Pro), and Griess reagent were purchased from Sigma-Aldrich (St. Louis, MO, USA). Halt protease and phosphatase inhibitor cocktail were purchased from Pierce Biotechnology (Rockford, IL, USA). The antibody against HO-1 was obtained from Santa Cruz Biotechnology (Santa Cruz, CA, USA); secondary anti-rabbit IgG (H and L, horseradish peroxidase-linked) was purchased from Cell Signaling Technology (Danvers, MA, USA). Anti-GAPDH was purchased from BioLegend Inc. (San Diego, CA, USA) and the anti-SOD1 and SOD2, GPx, catalase, and Nrf2 antibodies were from Abcam (Cambridge, UK). A multiple ELISA kit was obtained from Merck Millipore (Darmstadt, Germany). Zoletil was obtained from Virbac Laboratories (Fort Worth, Texas, USA). Novolink™ Polymer Detection System was purchased from Leica (Newcastle, UK). All other chemicals were of analytical grade, including Avicel PH 102 (FMC Biopolymer, USA) and Ludiflash (Pharma Ingredients & Services, Bishop, Texas, USA).

### 2.2. Preparation of PN Formula

The nine herbs constituting PN were purchased from a traditional medicine drugstore (Vejpong Pharmacy Co., Ltd., Bangkok, Thailand) on December 12^th^, 2013, and the herbs verified by Associate Professor Dr. Uthai Sotanaphun, Faculty of Pharmacy, Silpakorn University, Nakhon Pathom, Thailand. The voucher specimens (MUS1122-MUS1130) were deposited at the Museum of Natural Medicines, Faculty of Pharmaceutical Sciences, Chulalongkorn University, Bangkok, Thailand. A total of 180 kg of nine herbs in equal weight proportion (therefore 20 kg each) was grounded and macerated for 24 h in 80% ethanol using 10 times the total weight of crude materials. The crude product was extracted after subjecting it to treatment at 100°C for a duration of 3 h, after which the product was filtered before further evaporation to obtain the final herbal extract ratio at 3.3 : 1. A HPLC fingerprint of the PN extract containing gallic acid (3.4% w/w), vanillic acid (2.9% w/w), and ferulic acid (0.8% w/w) was previously described [11]. The extract was then mixed with Avicel PH 102 to produce an initial dry powder that was later bound together with Ludiflash. The final ratio of the PN extract : Avicel : Ludiflash compound was kept constant at 2 : 1 : 8 and was termed PN formula. For oral administration in rats, the PN formula was prepared in distilled water with a maximum volume of water at 2 mL/kg body weight (BW).

### 2.3. Animal Preparation

Male Sprague-Dawley rats (200–250 g) were obtained from the National Laboratory Animal Centre, Mahidol University, Thailand. The animals were housed in a temperature-controlled room under a 12-h light/dark cycle and were acclimatized for 1 week before starting the experiments. They had free access to water and standard diet. The body weight of each rat was measured daily in the morning. All procedures were approved by the Animal Research Ethics Committee of the Faculty of Medicine, Srinakharinwirot University, Bangkok, Thailand (Approval No. 14/2555 and 9/2556).

### 2.4. Experimental Protocols

ISO was prepared in normal saline and injected subcutaneously in the rats (5 mg/kg/day) for 2 consecutive days (days 26 and 27) at an interval of 24 h to induce IMI. Normal rats in groups 1 and 4–6 received injections of normal saline at a volume of 1 mL/kg. Body weights were measured before the injection. Animals were divided into nine groups (*n* = 5, each group) as follows:

  Group 1 (control): oroesophageal gavages of Avicel PH 102 : Ludiflash (1 : 4), 1000 mg/kg BW for 28 days in normal rats, used as a negative control
  Group 2 (IMI): oroesophageal gavages of Avicel PH 102 : Ludiflash (1 : 4), 1000 mg/kg BW for 28 days in IMI rats
  Group 3 (Pro + IMI): oroesophageal gavages of Pro, 30 mg/kg for 14 days before the end of experiment in IMI rats
  Group 4–6 (PN formula): oroesophageal gavages of PN formula (containing PN extract of 64, 127 or 255 mg/kg) for 28 days in normal rats
  Group 7–9 (PN formula + IMI): oroesophageal gavages of PN formula (containing PN extract of 64, 127 or 255 mg/kg) for 28 days in IMI rats.

At the end of the experiment, the rats were anesthetized with Zoletil.

### 2.5. Electrocardiography

To confirm the induction of MI in IMI rats, electrocardiography (ECG) was recorded after the second ISO injection (day 27) using a Cardiofax ECG 9620 (Nihon Kohden, Japan). After anesthesia, all rats were subjected to standard limb lead II recordings at a paper speed of 50 mm/sec and 1 mV = 1 cm calibration. All ST segments were calculated from the ECG recordings. Rats were immediately sacrificed by decapitation. Blood samples were collected and the hearts were then quickly removed for subsequent experiments.

### 2.6. Measurement of Cardiac Marker Troponin T

To confirm MI, troponin T, a cardiac marker, was also determined by electro-chemiluminescent immunoassays using a COBAS C800 (Roche Diagnostics, CL, USA).

### 2.7. Histopathological Examination

The hearts obtained from all the experimental groups were immediately fixed in 4% paraformaldehyde in phosphate-buffered saline, embedded in paraffin wax, and cut to 6 *μ*m thick cross sections. For histopathological studies, the sections were deparaffinized, rehydrated, and stained with hematoxylin and eosin. The sections were evaluated under a light microscope (Nikon, Eclipse E200, Nihon Kohden, Tokyo, Japan) and micrographs were obtained and analyzed using a Panoramic scanner and viewer software 1.15 (3D Histotech Ltd, Budapest, Hungary). The degree of histopathological changes of the heart was subjectively scored as follows: 0 (absence of myocardial necrosis, infiltration of inflammatory cells, and interstitial edema), 1 (mild lesion less than 1/3 of thickness of the heart wall), 2 (moderate lesion extends to 2/3 of thickness of the heart wall), 3 (severe lesion extends over 2/3 of thickness of the heart wall).

### 2.8. Measurement of NO Levels in Cardiac Tissues and Serum

To investigate the effect of PN on NO production, nitrite levels in cardiac tissues and serum were determined by the Griess reaction as described previously, though with slight modifications [12]. In brief, samples were deproteinized by zinc sulfate (15 mg/mL), shaken for 1 min, and centrifuged at 10000 g for 10 min. After centrifugation at 15000 g for 20 min, zinc sulfate (15 mg/mL) was added to the tissue homogenates and the mixture was shaken for 1 min and centrifuged at 15000 g for 20 min. Equal volumes of supernatant and Griess reagent (50 *μ*L) were mixed, transferred to 96-well microplates, and incubated for 30 min at 37°C. The absorbance of the reaction mixture was read at 540 nm using a microplate reader (Bio-Tex Synergy, Thermo Fisher Scientific, MA, USA). Sodium nitrate was used as a standard. The levels of NO in the cardiac tissues and serum are expressed as nM/mg protein.

### 2.9. Determination of TNF-*α* and IL-6 Levels in Serum

The pro-inflammatory mediators TNF-*α* and IL-6 levels in serum were measured by using a multiple ELISA kit (Merck Millipore, Darmstadt, Germany) according to the manufacturer's instructions. The absorbance was measured by a microplate reader. The levels of mediators are expressed as pg/mg of protein.

### 2.10. Western Blot Analysis

The protein expression of antioxidant enzymes was identified using immunoblotting. Briefly, whole proteins were extracted with ice-cold RIPA lysis buffer supplemented with Halt protease and phosphatase inhibitor cocktail (Pierce Biotech, IL, USA). The extracted protein was mixed with loading buffer (225 mM Tris–HCl, pH 6.8, 6% sodium dodecyl sulfate, 30% glycerol, 9% 2-mercaptoethanol, and 0.009% bromophenol blue) and incubated at 95°C for 5 min. An equivalent amount of proteins was electrophoresed on a 10% SDS-PAGE gel. The proteins were then transferred onto PVDF membranes (GE Healthcare, Buckinghamshire, UK). The membranes were blocked with 5% fat-free milk in TBST (10 mM Tris-HCl, pH 7.4, 0.1 M NaCl, and 0.01% Tween-20) for 1 h at room temperature, and then probed with the specific primary antibodies (1 : 1000) against catalase, HO-1, SOD1, SOD2, and GPx in 1–5% BSA diluted in TBST overnight at 4°C. GAPDH was used as an internal control to confirm equal loading of the samples. The membrane was further incubated with corresponding secondary antibodies coupled with horseradish peroxidase for 1 h at room temperature. The membranes were visualized by enhanced chemiluminescence (Merck Millipore, CA, USA), and photography using GeneGnome5 (Syngene, Cambridge, UK). The intensity of each protein band was quantified by ImageJ (NIH, Bethesda, MD, USA).

### 2.11. Immunohistochemistry of Nrf2 Expression

Tissue sections were deparaffinized and dehydrated through a graded alcohol series. Antigen retrieval was performed by boiling the sections in 0.01 M citrate buffer, pH 6.0 for 2 min and then maintained at a sub-boiling temperature for 10 min. Subsequently, 3% H_2_O_2_ in water for 30 min was used to quench the endogenous peroxidase activity. Sections were blocked in 5% BSA/TBST at 37°C for 30 min to eliminate non-specific binding, prior to incubation with primary antibodies against Nrf2 (1 : 10000 in 5% BSA) overnight at 4°C. According to protocol provided by the manufacturer, the sections were incubated with the Novalink TM Polymer Detection System, then incubated with diaminobenzidine to visualize the antibody-antigen complex and counterstained with hematoxylin for nuclear staining. The Nrf2-positive cells (brown nuclei) were viewed and counted in 6 random visual fields from each sample in all experimental groups using a Panoramic scanner and viewer software 1.15 (3D Histotech Ltd, Budapest, Hungary).

### 2.12. Statistical Analysis

Data are expressed as the mean ± SEM and were analyzed by GraphPad Prism version 6 (GraphPad Software, Inc., San Diego, CA, USA) using one-way analysis of variance (ANOVA) followed by Tukey's test. Except for the analysis of pathological score was analyzed with the corresponding non-parametric tests using the Kruskal-Wallis test with Dunn's Multiple Comparison Test, data were expressed as the mean ± SD. A *p*-value <0.05 was considered statistically significant.

## 3. Results

### 3.1. Effect of PN Formula on the Electrocardiogram

**Figure 1:**
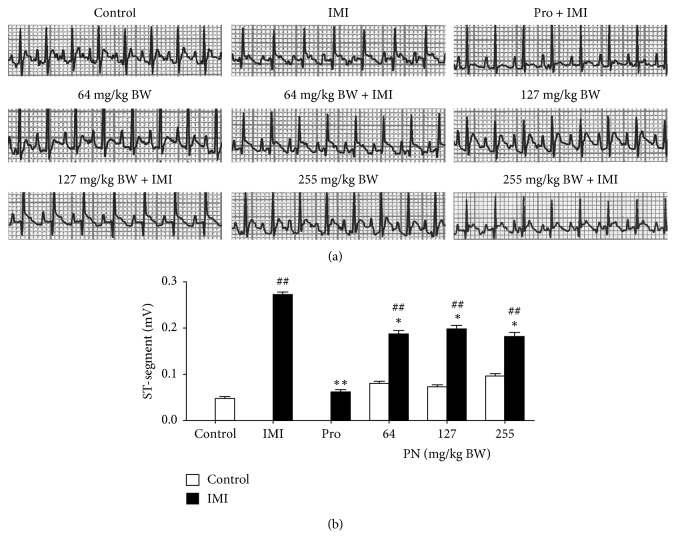
ECG recordings from the control, IMI, IMI pretreated with Pro (30 mg/kg), normal rats that received PN (64, 127, or 255 mg/kg), and IMI pretreated with PN (64, 127, or 255 mg/kg). (a) Representative ECG recordings and (b) bar graph of ST segment are shown. Values are the mean ± SEM (*n* = 5); speed: 50 mm/sec; amplitude: 1 mV/1 cm. ^##^*p* < 0.001 vs. the control. ^*∗*^*p* < 0.05 ^*∗∗*^*p* < 0.001 vs. the IMI rats.

The control group and the normal rats that received orally administered PN (64, 127, or 255 mg/kg) showed normal ECG readings (Figure 1(a)), whereas rats injected with ISO showed a significant increase in the ST segment, an indicator of MI, when compared with the controls. The administration of Pro (30 mg/kg) was able to statistically significantly dampen ISO-induced ST elevations when compared with the IMI rats (Figure 1(b)). Though PN formula treatment (at all doses) could not completely suppress the ISO-induced elevation of the ST segment, a significant decrease in the ST segment was observed in PN-treated rats compared with the IMI rats (Figure 1(b)).

### 3.2. Effect of PN Formula on Cardiac Marker Troponin T

**Figure 2:**
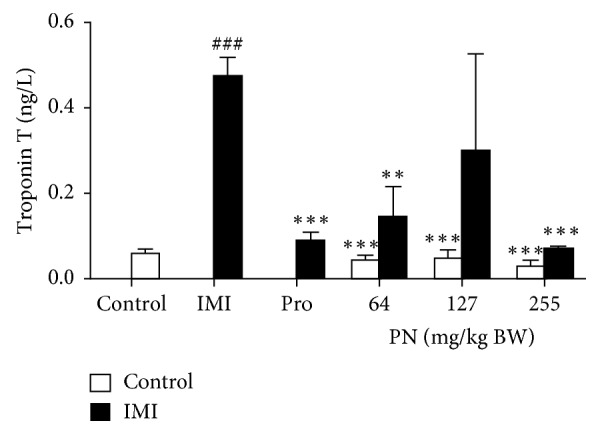
Serum cardiac troponin T level (ng/L) after PN administration (64, 127, or 225 mg/kg) and Pro (30 mg/kg) in normal and IMI rats. Values are presented as the mean ± SEM of four independent experiments. ^###^*p* < 0.0001 vs. the control. ^*∗∗*^*p* < 0.001 and ^*∗∗∗*^*p* < 0.0001 vs. the IMI rats.

The 28-day pre-treatment with PN formula did not significantly affect body weight, but serum troponin T was significantly increased (*p* < 0.0001) in IMI rats when compared to that of the control. PN (64 or 255 mg/kg) + IMI showed a significant decrease in troponin T (*p* < 0.001 and *p* < 0.0001, respectively) when compared to the IMI (Figure 2).

### 3.3. Effect of PN Formula on Histopathological Features

**Figure 3:**
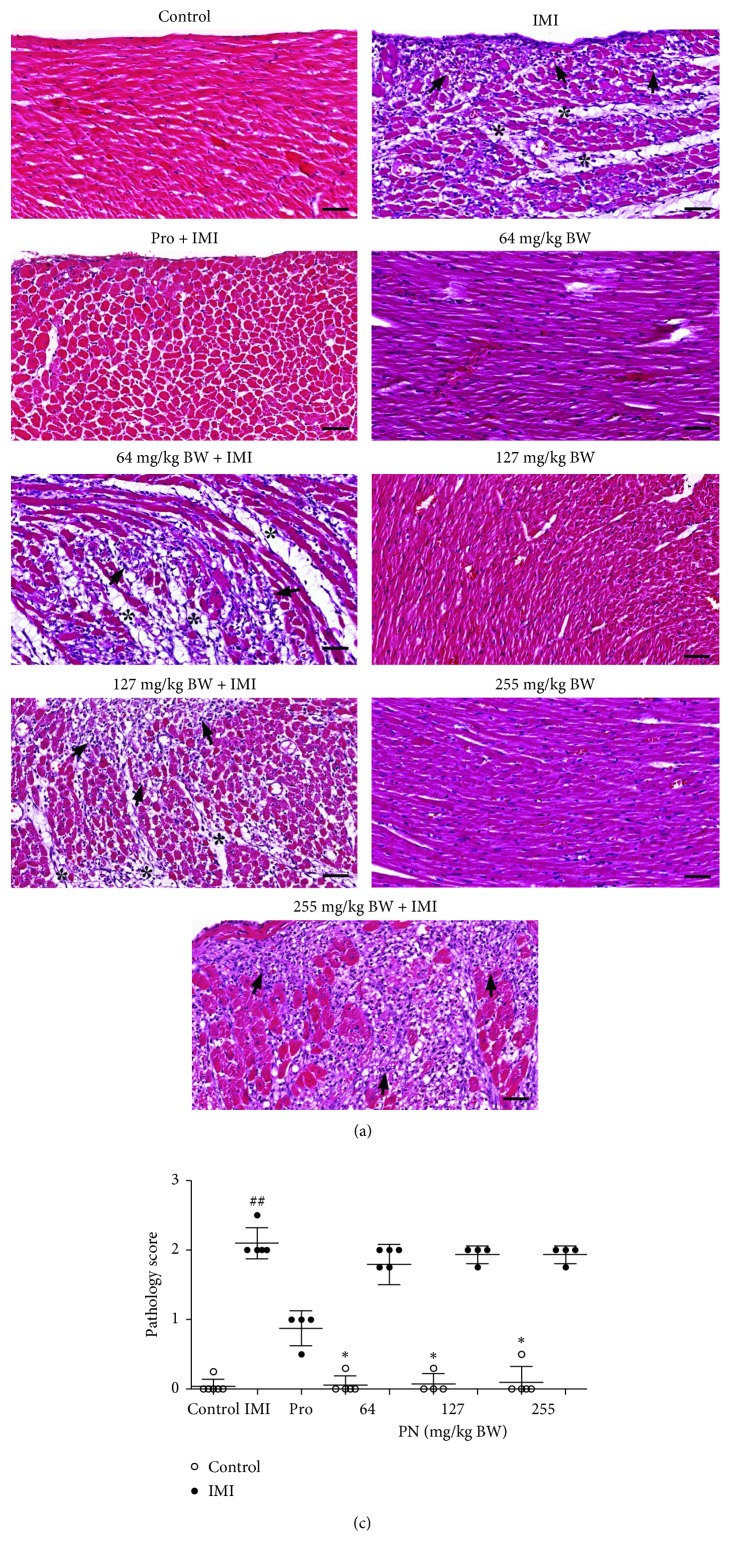
Effect of 28-day pretreatment with PN formula on histopathological changes. (a) Representative photomicrographs of cardiac tissues of the control, IMI, IMI pretreated with Pro, normal and IMI pretreated with PN (64, 127, or 255 mg/kg). IMI showed myocardial ischemia with inflammatory cell infiltration (⟶) and interstitial edema (^*∗*^). Scale bar: 50 *μ*m. (b) The degree of histopathological changes of the cardiac tissues has plotted a histogram. Values are expressed as mean ± SD (*n* = 5), ^##^*p* < 0.001 vs. the control. ^*∗*^*p* < 0.05 vs. the IMI rat.

The histopathological changes in the cardiac tissues of the rats after the oral administration of PN formula and IMI are shown in Figure 3(a) and quantified in Figure 3(b). The control group showed normally structured cardiac tissues. Normal rats that received PN (64, 127, or 255 mg/kg) showed slight migration of inflammatory cells in the left ventricle and septum compared with the controls. In contrast, the IMI rats apparently showed moderate to extensive myocardial necrosis and migration of inflammatory cells with interstitial edema in both septa of the left and right ventricles. However, 28-day pre-treatment with PN (all doses) before IMI induction was not able to reverse the ISO-induced histopathological alternations of the cardiac tissues back to normal.

### 3.4. Effect of PN Formula on NO Levels in Cardiac Tissue and Serum

**Figure 4:**
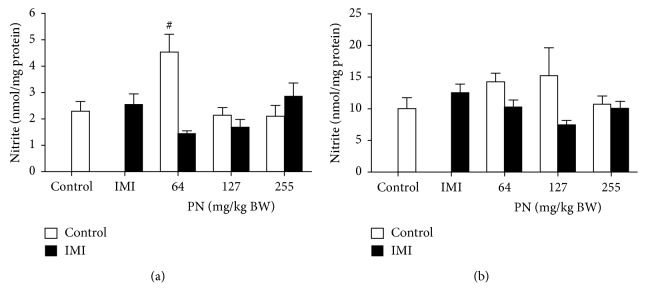
Effect of 28-day pretreatment with PN formula on NO levels in (a) cardiac tissues and (b) serum. Values are presented as the mean ± SEM of four independent experiments. ^#^*p* < 0.05 vs. control.

Cardiac NO was significantly increased (*p* < 0.05) only in normal rats that received PN at the lowest dose (64 mg/kg), compared with the controls (Figure 4(a)). There was no significant difference between any treatment group with regard to serum NO (Figure 4(b)).

### 3.5. Effect of PN Formula on Serum TNF-*α* and IL-6 Levels

**Figure 5:**
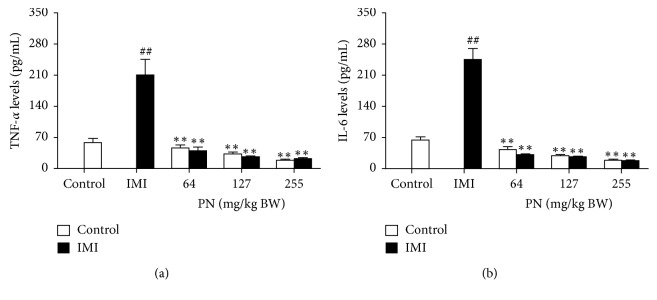
Effect of 28-day pretreatment with PN formula on (a) TNF-*α* and (b) IL-6 levels in serum. Values are presented as the mean ± SEM of four independent experiments. ^##^*p* < 0.001 vs. the control. ^*∗∗*^*p* < 0.001 vs. the IMI rats without the extract.

A subcutaneous injection of ISO (5 mg/kg) administered for two consecutive days significantly increased serum TNF-*α* and IL-6 levels (*p* < 0.001), compared with the controls (Figure 5). The oral administration of PN (64, 127, or 255 mg/kg) significantly decreased serum TNF-*α* and IL-6 levels (*p* < 0.001) in both the control and IMI rats in a dose-dependent manner.

### 3.6. Effect of PN Formula on Protein Expression of Antioxidant Enzymes in Cardiac Tissues

**Figure 6:**
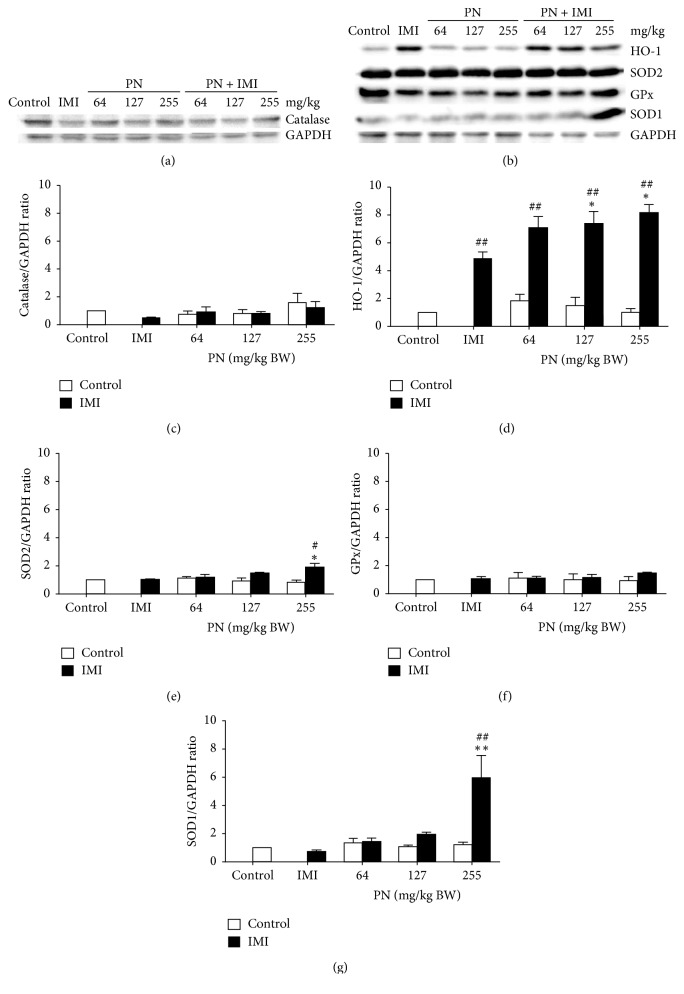
Effect of 28-day pretreatment with PN formula on antioxidant enzymes, HO-1, SOD1, SOD2, GPx, and catalase expression in cardiac tissues as evaluated by immunoblotting. (a) Representative protein bands of catalase. (b) Representative protein bands of HO-1, SOD2, GPx, and SOD1. Densitometric quantification of (c) catalase, (d) HO-1, (e) SOD2, (f) GPx, and (g) SOD1 are shown. Values are presented as the mean ± SEM of four independent experiments. ^#^*p* < 0.05, ^##^*p* < 0.001 vs. the control. ^*∗*^*p* < 0.05, ^*∗∗*^*p* < 0.001 vs. the IMI rats.

PN at the lowest dose (64 mg/kg) with ISO injection significantly increased the protein expression of HO-1, compared with the controls (Figure 6(d)). Furthermore, PN at all doses significantly increased the protein expression of HO-1 in IMI rats in a dose-dependent manner, compared with the controls (*p* < 0.001) and the IMI rats (*p* < 0.05). Interestingly, the administration of PN only at the highest dose (255 mg/kg) significantly augmented the protein expression of SOD1 (*p* < 0.001) (Figure 6(g)) and SOD2 (*p* < 0.05) (Figure 6(e)) in IMI rats, compared with the control and IMI rats. Nevertheless, alterations in the protein expression of other antioxidant enzymes, such as catalase (Figure 6(c)) and GPx (Figure 6(f)), were not observed.

### 3.7. Immunohistochemical Analysis of Nrf2 in Cardiac Tissues

**Figure 7:**
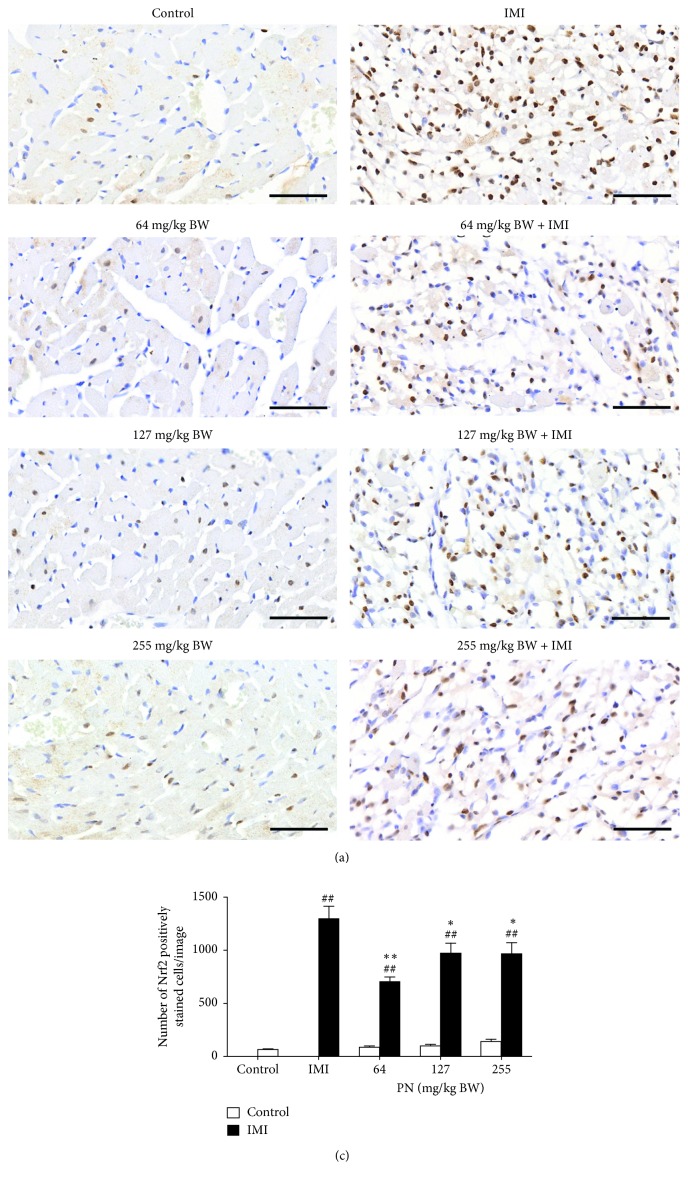
Effect of 28-day pretreatment with PN formula on Nrf2 content under light microscopy in rat cardiac tissues. (a) Representative images of cardiac tissues with immunohistochemistry of the control, IMI, normal and IMI pretreated with PN (64, 127, or 255 mg/kg) (magnification × 400). (b) Quantitative analysis results. Each column represents the mean ± SEM. ^##^*p* < 0.001 vs. the control. ^*∗*^*p* < 0.05, ^*∗∗*^*p* < 0.001 vs. the IMI rats.

The expression of Nrf2 in the control rats displayed slight light brown immunostaining in the cytoplasm and in the nuclei of myocardial tissue (Figure 7(a)). Significant Nrf2 expression (*p* < 0.001), as indicated by intense brown staining in the nuclei, was observed in IMI rats when compared with the control rats (Figure 7(b)). Significant differences were observed between IMI rats and IMI rats that received PN at 64 (*p* < 0.001), 127 (*p* < 0.05), and 255 mg/kg (*p* < 0.05). IMI rats that ingested PN had significantly upregulated (*p* < 0.001) Nrf2 expression when compared to the controls. No significant difference was observed between control rats and control rats that received PN (64, 127, or 255 mg/kg) (Figure 7(b)).

## 4. Discussion

Acute MI is one of the leading causes of morbidity and mortality throughout the world; its destructive effects on the heart have spurred research into alternative treatments that focus on cardioprotection. Therefore, the current study tested the cardioprotective effects on MI of a commonly used herbal formula, PN. Using rodent models of acute MI, the administration of ISO in this study led to a significant increase in ST elevation and troponin T with moderate to excessive myocardial necrosis and, infiltration of inflammatory cells with interstitial edema, thus confirming IMI, according to previously described experiments [3, 13]. The oral administration of Pro as a positive control reversed the effects of ISO on ECG patterns and troponin T level, further confirming the validity of the IMI rat model in the present study [14]. The long-term administration of PN formula (28 days) in IMI rats significantly dampened the elevation of the ST segment when compared with the IMI group, suggesting that PN formula had cardioprotective properties on MI-affected hearts. However, the 28-day administration of PN formula at all doses prior to the ischemia/reperfusion injury induced by ISO could not restore the ST segment, or the histopathological changes that occurred in the cardiac tissues to normal. In addition, administration of the compound increased NO in normal cardiac tissues, decreased the levels of the pro-inflammatory cytokines TNF-*α* and IL-6, increased the protein expression of SOD1, SOD2, and HO-1, and modulated Nrf2 expression.

The lowest dose of PN (64 mg/kg) significantly increased NO levels in cardiac tissue, but not in serum, in normal rats. NO, mainly generated from eNOS in endothelial cells and cardiomyocytes, plays an important role in myocardial function and vascular tone under physiological conditions by maintaining vasorelaxation tone [15, 16]. NO is one of alternative mechanisms underlying the pathophysiology of myocardial IR injury [9]. Under pathological conditions, the dysfunctional eNOS-NO pathway causes multiple cardiovascular diseases, including MI and coronary heart disease [7, 16]. A previous study showed that PN extract (100 *μ*g/mL)-induced vasorelaxation of aorta in an organ bath was partially inhibited by the concurrent pre-treatment of indomethacin and L-NAME [17]. In addition, eight weeks of exercise training in healthy male rats showed a significant decrease in myocardial infarct size from IR injury, which correlated with a significant increment of NO and its metabolites [18]. Present study, the 28-day administration of PN formula may not be sufficient to stimulate endogenous NO generation in IMI rats. It has been suggested that vascular NO preservation includes eNOS expression and activity and that the stability of NO is regulated by HO-1 expression [19].

TNF-*α* and IL-6 are crucial indicators of inflammation after MI in both mice and human [20] as well as in a rat model of IMI [1, 4, 7, 10, 14]. The observation that these mediators act as indicators of inflammation agreed with our IMI rat model in which a significant increase in the serum cytokine levels of TNF-*α* and IL-6 was observed. Even though the myocardial necrosis and inflammatory cell infiltrated observed by hematoxylin and eosin staining were not alleviated in PN-administered IMI rats, a dose-dependent decrease in the cytokine levels was found in both PN-administered normal and IMI rats. Therefore, our results suggest that PN might play an anti-inflammatory role through which it contributes to cardioprotection. As ISO is widely used to induce acute MI in rat models, it not only stimulates the release of pro-inflammatory cytokines, but also stimulates oxidative stress [1, 2, 4, 10, 21]. In addition, the alleviation of MI, as reflected by the reduction of TNF-*α* and IL-6 levels and ROS generation, correlates well with the silent information regulator 1-Nrf2-HO-1 pathway [22, 23].

HO-1 is induced by transcription factors, such as AP-1, Nrf2, and nuclear factor(NF)-*κ*B, in response to stress stimuli, including IR, oxidative stress, and inflammation [24]. It has been recognized that pro-inflammatory cytokines, including TNF-*α*, IL-1*β*, and IL-6, are potent inflammatory activators of NF-*κ*B, which is inhibited by Nrf2 as well as HO-1 [22, 23, 25]. There is a functional cross-talk between Nrf2 and NF-*κ*B transcription factors in response to oxidative stress and inflammation, respectively. Meanwhile, NF-*κ*B can modulate Nrf2 activation, and the absence of Nrf2 can cause an increment in NF-*κ*B activity leading to the stimulation of inflammation via the production of target cytokines [26]. In ISO-induced rats, the MI-activated survival proteins p-PI3K and p-Akt are involved in Nrf2 nuclear translocation and trigger an increase in HO-1 expression in myocardial tissue [6]. Similarly, MI induced by ISO caused an increase in HO-1 mRNA expression after 2 day of induction [27] as well as an increase in Nrf2 protein expression after 2 days of induction [28]. Meanwhile HO-1 deficiency exacerbates post-ischemic cardiac inflammation in mice, human HO-1 gene therapy showed cardioprotection by decreasing inflammation after ischemia injury performed by left anterior descending occlusion and reperfusion in murine and porcine hearts [29]. Protoporphyrin (PP) including SnPP and ZnPP, HO-1 inhibitors, has been used to demonstrate the effectiveness of substrates on cardioprotection against MI in rat models both *ex vivo* and *in vivo*, respectively [30, 31]. It has been reported that PP at pharmacological doses may have potential nonspecific targets in a biological system [32]. Therefore, the present study, which showed that pre-treatment with PN in IMI rats up-regulated the protein expression of HO-1, corroborates the findings of the experiments mentioned above, suggesting a relationship between MI and the Nrf2/HO-1 signaling pathway. This correlation is further supported by our findings that the oral administration of PN increased HO-1 expression in a dose-dependent manner, compared with both the control and IMI groups. The present study is concordant with a previous study that showed that oral pretreatment of PN for 7 days increased HO-1 expression in normal rats at the dose of 100 mg/kg [33] and in MI rats induced by left anterior descending coronary artery ligation at doses from 50–200 mg/kg [34]. It was shown that HO-1 and Nrf2 expression produces cardioprotection through anti-inflammatory and antioxidant effects [28]. Conversely, MI induced by ISO showed a reduction in the protein expression of HO-1 and Nrf2 while also decreasing the activity of glutathione, glutathione reductase, GPx, SOD, and catalase and increasing MDA [35, 36].

Previous studies demonstrated the protective effects of *Terminalia chebula* on ISO-induced oxidative stress through the restoration of the activities of antioxidant enzymes in heart tissues, including SOD, catalase, glutathione reductase, and GPx [37]. These redox-balancing proteins, which include HO-1, constitute part of a group of Nrf-targeted genes through the binding and activation of antioxidant response elements, leading to the production of antioxidant enzymes [38]. This understanding corresponds well to the results of the present study that showed that SOD1 and SOD2 levels, but not GPx or catalase, were significantly elevated in IMI rats that received only the highest dose of PN. These results are also supported by recent studies that showed that the antioxidant enzyme SOD1 or CuZn-SOD is the main cytoplasmic ROS scavenger, whereas SOD2 or Mn-SOD plays an important role against ROS in mitochondrial spaces [39]. Thus, high HO-1 protein expression may be able to induce or at least correlate with an increase in antioxidant enzyme levels, such as SOD, which is a first line of defense against oxidative stress by removing superoxide radicals in the heart.

Previous studies also demonstrated that PN extracts showed strong scavenging activity of superoxide anions, hydroxyl radicals, and nitric oxide [8] and showed anti-oxidative stress activity against hydrogen peroxide (H_2_O_2_)-induced oxidative stress in human endothelial ECV304 cells and yeast cells [8, 40]. The capacities of PN against the induction of antioxidant enzymes and inhibition of pro-inflammatory expression may be due to particular active ingredients, such as gallic acid, vanillic acid, and ferulic acid, which were used as chemical markers, as presented in previous studies. Pretreatment of gallic acid as well as vanillic acid in ischemia/reperfusion experiments has been able to increase antioxidant enzyme SOD, catalase, and GPx activities in isolated rat hearts [41, 42]. Administration of gallic acid, as a positive control, decreased MDA levels, increased the content of the antioxidant enzymes catalase and GPx, and alleviated histopathological changes, including edema, myonecrosis, and infiltration of inflammatory cells in the myocardium in IMI rats [43]. Vanillic acid, as a potent free radical scavenger, has been shown to inhibit the expression of proinflammatory cytokine genes (IL-1*β*, IL-6, and TNF-*α*) without necrosis or inflammatory cell infiltration in IMI rats [13] as well as a decrease in infarct size and MDA level in IR isolated rat heart [44]. Ferulic acid has also exhibited cardioprotective activity in IMI rats by decreasing lipid peroxidation and increasing the activities of antioxidant enzymes, including SOD, and glutathione [45].

As shown through the present study, the 28-day administration of PN did not appear to cause detrimental health effects in experimental rats. Nonetheless, caution may be required for the longer-term administration of high doses that exceed those used in the present study as it was reported that the oral administration of PN extract at 1000 mg/kg or the daily ingestion of PN extract at 100 mg/kg for 90 days significantly increased BUN in male rats and blood triglyceride levels in female rats [46]. Oral administration of PN at a high dose (1000 mg/kg/day) for 12 months did not cause any remarkable toxic effects, however relatively toxic associated with mesangiolysis in both male and female Sprague Dawley rats [47]. With proper dosage and administration, PN could be further developed to be used as an alternative herbal remedy or as an adjunct to other therapeutic agents in order to take advantage of its anti-inflammatory and/or antioxidant activities.

## 5. Conclusion

The current study appears to be the first to establish the anti-inflammatory activities of the ethanol extract of PN in IMI rats. Long-term administration (28 days) of PN formula in IMI rats decreased serum levels of the pro-inflammatory cytokines TNF-*α* and IL-6, up-regulated the protein expression of SOD1, SOD2, and HO-1, and moderated Nrf2 expression. PN formula also increased NO production in cardiac tissues in normal rats. Our results reveal that the cardioprotective role of PN against IMI involved in the Nrf2/HO-1-mediated induction of antioxidant enzymes. PN was therefore demonstrated to be a promising herbal formula for the protection and/or alleviation of inflammation as well as oxidative stress in MI induced by ISO.

## Data Availability

The data used to support the findings of this study are available from the corresponding author upon request.
